# Effects of kolaviron on hepatic oxidative stress in streptozotocin induced diabetes

**DOI:** 10.1186/s12906-015-0760-y

**Published:** 2015-07-16

**Authors:** Omolola R. Oyenihi, Nicole L. Brooks, Oluwafemi O. Oguntibeju

**Affiliations:** Department of Biochemistry, Bowen University, Iwo, Nigeria; Department of Wellness Sciences, Cape Peninsula University of Technology, Cape Town, South Africa; Nutrition and Chronic Diseases Unit, Oxidative Stress Research Centre, Department of Biomedical Sciences, Cape Peninsula University of Technology, Bellville, South Africa

**Keywords:** Diabetes, Antioxidant, Apoptosis, Liver

## Abstract

**Background:**

Alteration in antioxidant defence and increase in oxidative stress that results in tissue injury is characteristic of diabetes. We evaluated the protective effects of kolaviron (a flavonoid complex extracted from the seeds of *Garcinia kola*) on hepatic antioxidants, lipid peroxidation and apoptosis in diabetic rats.

**Methods:**

To induce diabetes, rats were injected with streptozotocin intraperitoneally at a single dose of 50 mg/kg. Kolaviron (100 mg/kg) was administered orally for 6 weeks (5 times weekly). Activities of liver antioxidant enzymes was analysed with Multiskan Spectrum plate reader. High performance liquid chromatography (HPLC) was used in the analysis of MDA (malondialdehyde), a product of lipid peroxidation. Apoptosis was assessed by terminal deoxynucleotidyl transferase-mediated dUTP nick end labeling (TUNEL) assay.

**Result:**

Diabetic rats exhibited a significant increase in the peroxidation of hepatic lipids as observed from the elevated level of malondialdehyde (MDA). In addition, Oxygen Radical Absorbance Capacity (ORAC), level of reduced glutathione (GSH), ratio of reduced to oxidized glutathione (GSH: GSSG) and catalase (CAT) activity were decreased in the liver of diabetic rats. The activities of GPX (glutathione peroxidase) and SOD (superoxide dismutase) were unaltered in diabetic rats. TUNEL assay revealed increased apoptotic cell death in the liver. Kolaviron attenuated lipid peroxidation and apoptosis, increased CAT activity, GSH levels and GSH: GSSG ratio. The ORAC of kolaviron-treated diabetic liver was restored to near-normal values.

**Conclusion:**

Kolaviron protects the liver against oxidative and apoptotic damage induced by hyperglycemia.

## Background

Diabetic patients are more predisposed to microvascular and macrovascular complications. Although the control of blood glucose and dyslipidaemia is beneficial, these therapeutic approaches cannot reverse organ damage. Reports have shown that chronic generation of reactive oxygen species (ROS) due to hyperglycemia play a critical role in the development of diabetic liver injury, hence the use of antioxidant rich plant materials in the control of diabetes has received considerable attention [1, 2]. Due to the involvement of oxidative stress in diabetes, antioxidants might attenuate or delay the hepatic injury in diabetic patients and in experimental models. The pharmacological effects of kolaviron (KV), a flavonoid complex isolated from the *Garcinia kola* seed (also known as bitter kola) in animal models are extensive, ranging from protection against *Plasmodium berghei* infection and hepatoxicity by toxins such as carbon tetrachloride [3] to glucose lowering effect [4]. The active compounds so far reported in KV includes *Garcinia* biflavonoid (GB) 1, GB-2, kolaflavanone, kolaflavone and binaringenin [5, 6]. KV has been shown to reduce oxidative stress in many studies [7, 8]. In regard to the central role of oxidative stress in the pathogenesis of diabetic liver injury, this study was designed to evaluate the effect of KV on oxidative stress and apoptosis in the liver of streptozotocin-induced diabetic rats.

## Methods

### Animals

The study protocol was approved by the Faculty of Health and Wellness Sciences Research Ethics Committee of the Cape Peninsula University of Technology (Ethics Certificate no: CPUT/HW-REC 2012/AO4). All received humane care in accordance to the criteria outlined in the ‘Guide for the Care and Use of Laboratory Animals’ prepared by the National Academy of Science (NAS) and published by the National Institute of Health (Publication no. 80–23, revised 1978). Male Wistar rats (11–12 weeks, 270 ± 25 g), were used for the study. Experiments were performed at the animal facility of the Medical Research Council (MRC) and strictly adhered to the standard operating procedures (SOPs). All animals were housed individually at room temperature (22 ± 2 °C) with 55 ± 5 % humidity and an automatically controlled cycle of 12 h light and 12 h dark. A standard laboratory diet and water were provided *ad libitum* and rats were habituated to the experimental conditions 1 week prior to experimentation.

### Plant materials

Fresh seeds of *Garcinia kola* were purchased from Bodija market in Ibadan, Oyo State, Nigeria and authenticated by Professor E. A. Ayodele at the Department of Botany, University of Ibadan. A voucher specimen (FHI-109777) is available at the herbarium of the Forestry Research Institute of Nigeria (FRIN), Ibadan.

### Extraction of kolaviron (KV)

*Garcinia kola* seeds were peeled, sliced and air-dried (25–28 °C). KV was isolated according to the method of Iwu et al. [4]. The powdered seeds (600 g) were defatted with 800 ml of light petroleum ether (bp 40–60 °C) in a soxhlet for 24 h. The defatted powder was spread in thin layers on trays and air dried at room temperature for 24 h, repacked in the soxhlet and extracted with acetone (500 ml) at a temperature of 40 °C. The extract was concentrated and diluted twice its volume with distilled water and extracted with ethylacetate (6 × 300 ml). The concentrated ethylacetate yielded KV (12 g).

### Induction of diabetes

Diabetes was induced in rats by a single intraperitoneal injection of freshly prepared solution of 50 mg/kg streptozotocin (Sigma-Aldrich, Johannesburg, SA) in citrate buffer (0.1 M, pH 4.5) to overnight fasted rats. Diabetes was confirmed by stable hyperglycemia (>18 mmol/l) in the tail blood glucose after 5 days of streptozotocin (STZ) injection using a portable glucometer (Accu-Chek, Roche, Germany).

### Study design and tissue collection

The animals were divided into 4 groups (*n* = 10 per group): Normal control (C group), KV treated normal control (C + KV), diabetic control (D group) and KV-treated diabetic group (D + KV group). Treatment was started on the 6th day post STZ injection and continued for 6 weeks. KV (100 mg/kg b.wt.), dissolved in dimethylsulphoxide (DMSO: 100 %) to a final concentration of 70 mg/ml, was administered by gastric gavage 5 times a week. 100 mg/kg of KV was a more effective dose among the doses (100 and 200 mg/kg) investigated in our preliminary study. The dosage of KV was adjusted every week according to any change in body weight to maintain similar dose over the period of study. At the end of experimental period, The liver was rapidly excised, washed in ice-cold phosphate buffered saline, blotted, frozen in liquid nitrogen, and stored at −80 °C for biochemical estimations.

### Oxygen Radical Absorbance Capacity (ORAC)

The ORAC assay was conducted to kinetically measure the peroxyl radical scavenging activity in liver samples with trolox (6-hydroxy-2,5,7,8-tetramethylchroman-2-carboxylic acid) as the antioxidant standard according to the method of Ou et al. [9]. Liver homogenates were deproteinized with 0.5 M perchloric acid (1:1, v/v) and centrifuged at 10,000 g for 10 min. The supernatant was stored at −80 °C prior to analysis. Fluorescein (FL) was used as the fluorescent probe and the peroxyl radicals were generated from AAPH (2,2′-azobis (2-methylpropionamidine) dihydrochloride) in 75 mM phosphate buffer (pH 7.4). Specifically, 138 μL of 14 μM FL solution was mixed with 12 μL of diluted sample (1:20) with 75 Mm phosphate buffer, pH 7.4) standard, or blank (phosphate buffer, pH 7.4) to a black 96-well flat bottom plate and the plate was incubated at 37 °C for 20 min. After incubation, the reaction was started by the addition of 50 μL of AAPH (4.8 mM) to the mixture. Standards and samples were measured in triplicate. The fluorescence of the reaction mixture was monitored and recorded every minute (excitation = 485 nm and emmission = 535 nm) for 2 h with a Fluoroscan Ascent plate reader (Thermo Fischer Scientific, Waltham, MA, USA). Results were determined by using a regression equation relating trolox concentrations and the net area under the kinetic fluorescein decay curve (*y = ax2 + bx + c*). The ORAC value was expressed in micromoles of trolox equivalents per gram of tissue (μmol TE/g).

### Estimation of superoxide dismutase activity

Superoxide dismutase (SOD) was determined by the method of Crosti et al. [10]. The reaction mixture in a 96-well plate consisted of 15 μL of sample, 170 μL of 0.1 mM DETAPAC (Diethylenetriaminepentaacetic Acid) in 50 Mm sodium phosphate buffer (pH 7.4), and 20 μL of 1.6 mM 6-hydroxydopamine which initiated the reaction. The reaction was measured at 490 nm for 4 min at 30 s intervals and SOD activity expressed as U/mg of protein.

### Estimation of glutathione peroxidase activity

Activities of antioxidant enzymes were determined in a clear 96-well plate using a Multiskan Spectrum plate reader (Thermo Fisher Scientific, USA). Glutathione peroxidase (GPx) activity was determined according to the method of Ellerby & Bredesen [11]. To initiate the reaction, 25 μL of H_2_O_2_ (15 mM) was added to a final reaction mixture containing 2.5 μL of GSH (0.1 M), 2.5 μL of GR (0.1 U/mL), 5 μL liver homogenate, 5 μL of NADPH (15 mM in 0.1 % NaHCO3), 2.5 μL of sodium azide (100 mM) and 210 μL of assay buffer (50 mM potassium phosphate, 1 mM EDTA (Ethylenediaminetetraacetic acid), pH 7.0. The rate of H_2_O_2_-dependent oxidation of NADPH was monitored at 340 nm at 30 s intervals for 2 min. The activity of GPx was calculated using the extinction coefficient of 6.22 mM-1 cm-1 and results expressed as nmol NADPH oxidized per min per μg protein.

### Estimation of catalase activity

Catalase (CAT) activity was determined by the method of Aebi [12]. The assay is based on the principle of measurement of decomposition of hydrogen peroxide (H_2_O_2_) by catalase measured at 240 nm. Assay mixture contained 5 μL of sample, 170 μL of 50 mM potassium phosphate (pH 7.0) and 50 μL of 0.1 % hydrogen peroxide in 50 mM potassium phosphate (pH 7.0). The rate of decomposition of H_2_O_2_ was measured at 240 nm for 2 min in 15 s intervals in a Multiskan Spectrum plate reader (Thermo Fisher Scientific, USA). Catalase activity is expressed as mol H_2_O_2_ consumed/min/mg protein.

### Glutathione status analysis

Glutathione (GSH and GSSG) status was determined according to the method of Asensi et al. [13]. Liver samples were homogenized (1: 10) in 6 % (*v/v*) Perchloric acid (PCA) containing 1 mM EDTA for reduced glutathione (GSH) determination and for oxidized glutathione (GSSG) determination, liver samples were homogenized in 6 % PCA containing freshly prepared 3 Mm M2VP (1-methyl-2-vinylpyridinium trifluoromethanesulfonate) and 1 Mm EDTA (Ethylenediaminetetraacetic acid). Homogenates were centrifuged at 15,000 g for 5 min, 50 μL of supernatant was added to 50 μL of 0.3 mM DTNB (5,5′-dithiobis-2-nitrobenzoic acid) and 50 μL of glutathione reductase (1U). The reaction was initiated by addition of 50 μL of 1 mM NADPH and change in absorbance was monitored at 410 nm for 5 min. GSH and GSSG Levels were calculated using GSH and GSSG as standards.

### Estimation of lipid peroxidation

Liver malondialdehyde (MDA) was determined by HPLC using a method adapted from Khoschsorur et al. [14]. Briefly, 100 μL of liver homogenates and standard were mixed with 750 μL orthophosphoric acid (0.44 M), 250 μL of 42 mM aqueous TBA (thiobarbituric acid) and 450 μL distilled water. The mixture was heated in a boiling water bath for 60 min. After cooling on ice, alkaline methanol (50 ml methanol + 4.5 ml 1 M NaOH) was added (1:1). The samples were centrifuged at 3500 g for 3 min at 4 °C. 1 mL of supernatant was added to 500 μL of n-hexane and the mixture centrifuged at 14,000 g for 40 s. 50 μL of the supernatant was then chromatographed on an Agilent 1200 series HPLC. A 5 μm YMC-PackPro C18 (1 5 0 m m × 4 . 6 m m i.d.) column was used for separation with 60:40 (*v/v*) 50 mM phosphate buffer, pH 6.8-methanol as mobile phase. The flow rate was 1 mL min − 1. Fluorometric detection was performed with excitation at 532 nm and emission at 552 nm. The peak of the MDA-TBA adduct was calibrated with the MDA standard.

### Terminal deoxynucleotidyl transferase-mediated dUTP nick end labeling (TUNEL) assay

For detection of apoptotic cells, liver sections were stained with the reagents supplied by ApopTag fluorescein. In situ Apoptosis Detection Kit (Chemicon, Billerica, CA). Briefly, each slide was deparaffinized, rehydrated, and treated with proteinase K (20 mg/L) for 15 min. Equilibration buffer was applied directly on the slides followed by incubation with the TUNEL reaction mixture containing terminal deoxynucleotidyl transferase (TdT) and digoxigenin nucleotide and unlabeled nucleotide for 1 h in a humidified chamber at 37 °C. Sections were counter stained with a mounting medium containing 0.5 μg/mL of propidium iodide and viewed by Olympus IX-81 microscope. Apoptotic cell death was quantitatively analyzed by counting TUNEL positive cells selected randomly from five consecutive fields at ×10 using the image analysis software ‘ImageJ’. TUNEL positive cells were expressed as percentage of total cells.

### Statistical analysis

Data were expressed as the means ± standard deviation. Significant differences between mean values of different groups were determined by one-way analysis of variance (ANOVA) with MedCalc software. Data not normally distributed was log transformed and analyzed using the Kruskal–Wallis one-way ANOVA on ranks hypotheses. Differences were considered significant at *p* < 0.05.

## Results

**Fig. 1:**
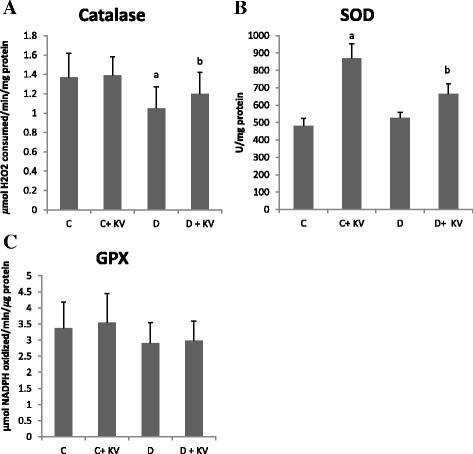
**a**–**c** Effects of KV on the activities of antioxidant enzymes; catalase (CAT), superoxide dismutase (SOD) and glutathione peroxidase (GPX) in the hepatic tissues of experimental rats. Data are presented as mean ± S.D. *a* Values differ significantly from those of control (*p* < 0.05). *b* Values differ significantly from diabetic group (*p* < 0.05). *C* Non-diabetic control rats, *C + KV* kolaviron-treated control rats, *D* untreated diabetic rats, *D + KV* kolaviron-treated diabetic rats

**Fig. 2:**
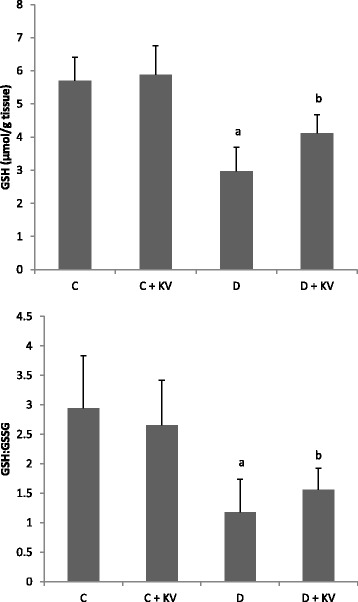
Effects of KV on GSH levels and GSH/GSSG ratio in hepatic tissues of experimental rats. Data are presented as mean ± S.D. *a* Values differ significantly from those of control (*p* < 0.05). *b* Values differ significantly from diabetic group (*p* < 0.05). *C* Non-diabetic control rats, *C + KV* kolaviron-treated control rats, *D* untreated diabetic rats, *D + KV* kolaviron-treated diabetic rats

**Fig. 3:**
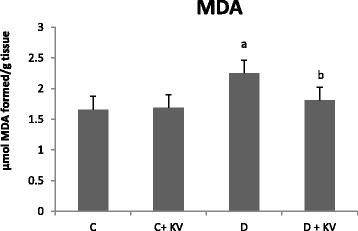
Effects of KV on malondialdehyde (MDA) concentration in hepatic tissues of experimental rats. Data are presented as mean ± S.D. *a* Values differ significantly from those of control (*p* < 0.05). *b* Values differ significantly from diabetic group (*p* < 0.05). *C* Non-diabetic control rats, *C + KV* kolaviron-treated control rats, *D* untreated diabetic rats, *D + KV* kolaviron-treated diabetic rats

**Fig. 4:**
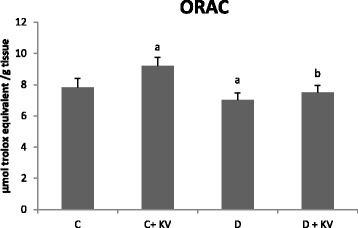
Effects of KV on oxygen radical absorbance capacity (ORAC) in hepatic tissues of experimental rats. Data are presented as mean ± S.D. *a* Values differ significantly from those of control (*p* < 0.05). *b* Values differ significantly from diabetic group (*p* < 0.05). *C* Non-diabetic control rats, *C + KV* kolaviron-treated control rats, *D* untreated diabetic rats, *D + KV* kolaviron-treated diabetic rats

The effects of KV on antioxidant status of experimental rats are shown in Figs. 1, 2, 3, 4. Although no change was observed in the activities of glutathione peroxidase and superoxide dismutase, catalase activity was decreased in diabetic tissues and normalized following KV administration (Fig. 1a–c). However, KV increased activity of superoxide dismutase in diabetic rats. Oxygen radical absorbing capacity (ORAC) was reduced significantly in diabetic rats but elevated after KV administration (Fig. 4). KV also boosted ORAC in normal rats. GSH level and GSH/GSSG ratio decreased in hepatic tissues of diabetic rats (Fig. 2). Treatment with KV improved these alterations as observed from increased levels of GSH and GSH/GSSG ratio in comparison to diabetic control group. The concentration of lipid peroxidation marker, malondialdehyde (MDA) was significantly (*P* < 0.05) higher in diabetic control group. Treatment of diabetic rats with KV decreased hepatic MDA concentration (Fig. 3).

**Fig. 5:**
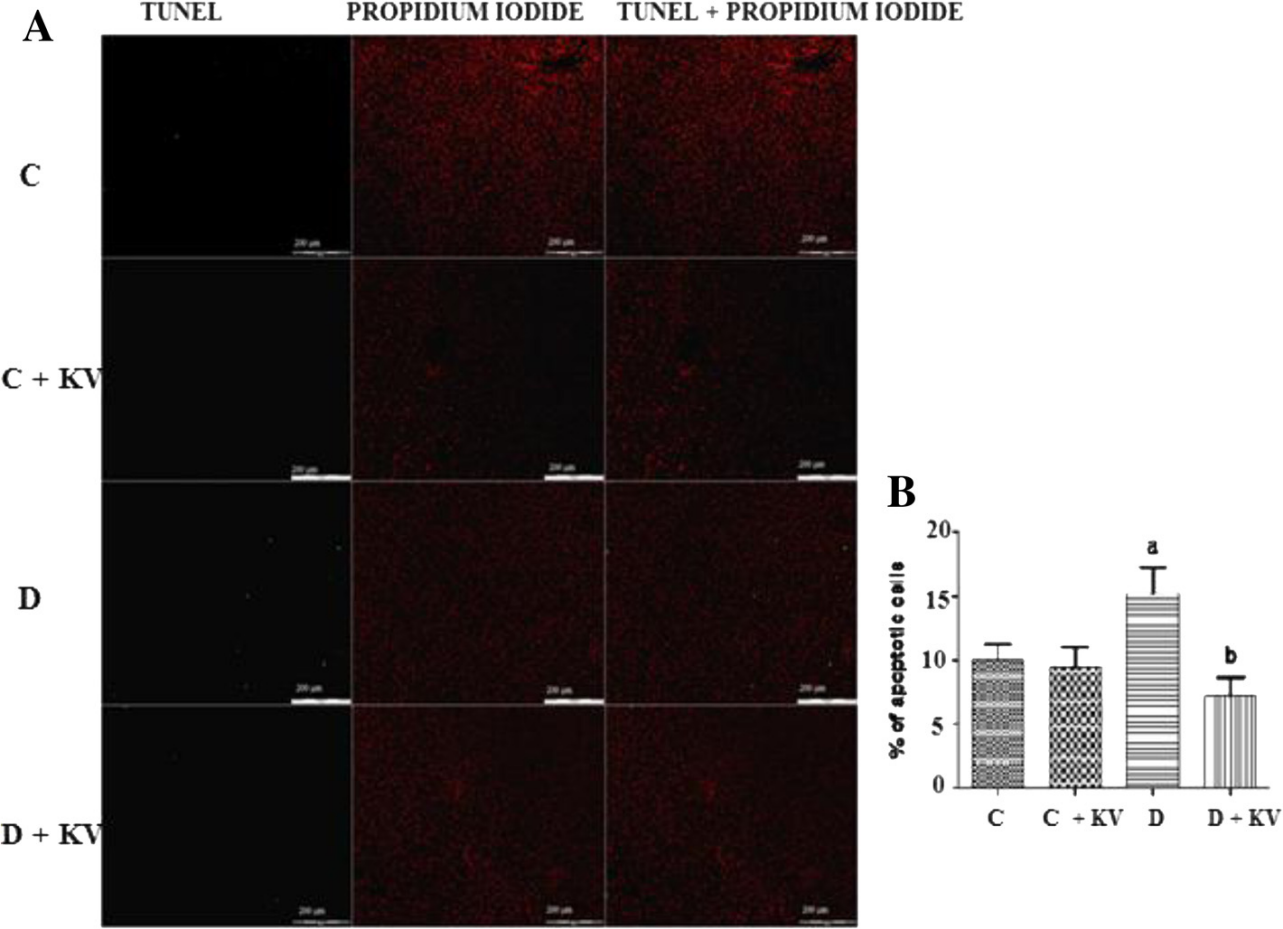
**a** and **b** Effects of KV on apoptosis in the hepatic tissues of experimental rats. Data are presented as mean ± S.D. *a* Values differ significantly from those of control (*p* < 0.05). *b* Values differ significantly from diabetic group (*p* < 0.05). *C* Non-diabetic control rats, *C + KV* kolaviron-treated control rats, *D* untreated diabetic rats, *D + KV* kolaviron-treated diabetic rats

Examination of hepatic apoptosis with TUNEL staining revealed an increase of TUNEL positive cells in the liver sections of diabetes control group (Fig. 5). KV reduced apoptotic cells in the liver of diabetic rats.

## Discussion

Oxidative stress–an imbalance between the generation of reactive oxygen species (ROS) and the compensatory response from the endogenous antioxidant network, has been demonstrated to play a pivotal role in diabetic vascular complications [15]. Therefore, it has been proposed that scavengers of oxidative stress may have a positive effect in alleviating diabetes as well as reduce its secondary complications. Antioxidants like superoxide dismutase (SOD), catalase (CAT), glutathione peroxidase (GPX) and reduced glutathione (GSH) effectively forms a defensive alliance against the onslaught of ROS protecting cells from oxidative damage. Reports have shown variation in the activities of antioxidant enzymes in diabetic rats. Although reductions of enzyme activities have been reported, some studies also demonstrated an increase and no change in enzyme activities. Possible reasons for the contradictory reports are variation in disease severity, duration, tissue specificity or other experimental conditions [16].

The enzymatic antioxidant-catalase (CAT) is involved in the removal of hydrogen peroxide in living cells and protects against hydroxyl radicals toxicity. In the present study, a significant decrease in CAT activity in diabetic rats may reflect the inability of the liver to eliminate hydrogen peroxide, a ROS. KV treatment enhanced CAT activities, demonstrating the antioxidant and tissue protective effects of KV.

Activities of SOD and GPX were not significantly different in diabetic controls compared to non-diabetic rats. KV increased SOD activity in both normal and diabetic rats. Superoxide dismutase (SOD) is an important antioxidant defence that catalyse the breakdown of superoxide radical anion. The increase activity of SOD in diabetic and non-diabetic rats after KV treatment might be a mechanism to boost antioxidant defence. There is a report of increased mRNA expression of SOD in cultured interstitial Leydig cells (ILCs) in the presence of KV [7]. The increase activity of SOD was accompanied by an increase in oxygen radical absorbing capacity (ORAC), further supporting the protective role of KV against free radicals.

Glutathione is the most abundant non enzymatic intracellular antioxidant [17]. Depletion of reduced Glutathione (GSH) either by conjugation and removal from the cell or oxidation to GSSG could significantly affect the overall redox potential of the cell [18]. The beneficial role of glutathione as an antioxidant depends not only on the glutathione pool size but also on its reduction/oxidation status [19]. The ratio of GSH to GSSG is a sensitive indicator of oxidative stress. In the present study, diabetic state resulted in a significant depletion of GSH level and GSH/GSSG ratio indicating its increased utilization against reactive oxygen species generated in diabetic rats. An elevation of the GSH stores and GSH/GSSG ratio in the liver of diabetic rats treated with KV compared to diabetic control suggests the alleviation of free radical damage.

An elevated level of liver MDA, a product of lipid peroxidation, has been reported in diabetic rats [20–23]. Results from our study corroborate these observations. KV treatment of diabetic rats reduced lipid peroxidation indicating its free radical scavenging property.

Apoptosis, a programmed cell death although occurs normally to maintain tissue homeostasis, it can become uncontrolled or dysregulated leading to deleterious pathological consequences such as diabetes [24]. Several studies have shown a positive relationship between hyperglycemia-induced oxidative stress and apoptosis [25–28]. Jaeschke et al. also demonstrates that the diabetic state increased oxidative stress and cell death of hepatocytes and endothelial cells [29]. Similarly, our work corroborates these observations as apoptosis was significantly increased in the liver post-streptozotocin injection. Treatment with KV however, protected hepatic cells from apoptotic death suggesting the anti-apoptotic property of KV.

## Conclusions

The present study explored the effect of KV on oxidative stress and diabetic liver injury and the resultsdemonstrated that KV treatment of diabetic rats protected against hyperglycemia-induced apoptosis andpromoted survival of hepatocytes, perhaps by scavenging free radicals.
